# Adverse effects of systemic advanced melanoma therapies—do BRAF/MEK inhibitors increase the incidence of mesenteric panniculitis?

**DOI:** 10.1007/s00330-025-11642-w

**Published:** 2025-05-01

**Authors:** Marcel Alexander Drews, Alexander Baumgarten, Sebastian Zensen, Marcel Opitz, Denise Bos, Lisa Zimmer, Selma Ugurel, Johannes Haubold, Dirk Schadendorf, Elisabeth Livingstone, Benedikt M. Schaarschmidt

**Affiliations:** 1 Institute of Diagnostic and Interventional Radiology and Neuroradiology, University Hospital Essen, Essen, Germany; 2 Department of Dermatology, University Hospital Essen, Essen, Germany

**Keywords:** Melanoma, Mesenteric panniculitis, BRAF/MEK, Immunotherapy, Side effects

## Abstract

**Objectives:**

BRAF/MEK inhibitors (BRAFi/MEKi) and PD-1 and CTLA-4 immune checkpoint inhibitors (ICI) have revolutionized malignant melanoma treatment and improved patients’ clinical outcome significantly. However, these therapies are associated with substance class-specific side effects. Here, selected cases indicate a correlation between the incidence of mesenteric panniculitis (MP) and BRAFi/MEKi treatment. As MP can mimic or conceal underlying malignancy, the aim of the present study was to confirm a potential correlation with BRAFi/MEKi or ICI in a retrospective, observational analysis of melanoma patients.

**Materials and methods:**

In a monocentric retrospective study, abdominal CTs of 490 melanoma patients receiving first-line treatment with ICI (nivolumab, ipilimumab, pembrolizumab, nivolumab/ipilimumab) or BRAFi/MEKi (dabrafenib/trametinib, vemurafenib/cobimetinib, encorafenib/binimetinib) in the adjuvant or advanced situation were evaluated for MP development comparing baseline imaging prior therapy start and follow-up imaging under therapy. MP was defined as an unilocular mesenteric mass characterized by small tissue nodules with increased density of the adjacent fat and a surrounding pseudo-capsule.

**Results:**

384 melanoma patients with ICI (161 women, median age at therapy start: 62 years, IQR: 21 years) and 106 patients with BRAFi/MEKi first-line therapy (46 women, median age: 58 years, IQR: 18 years) were evaluated. MP incidence was significantly higher following BRAFi/MEKi treatment compared to ICI (7.5% vs. 2.9%, *p* = 0.04). No significance was detected comparing time until MP development from therapy start (174 days, IQR: 518 days [BRAFi/MEKi] vs. 207 days, IQR: 298 days [ICI], *p* > 0.05).

**Conclusion:**

Our study demonstrates a significant increase in MP development following BRAFi/MEKi treatment compared to ICI in patients with melanoma. As this benign condition can mimic or even conceal malignancy, awareness of its appearance is important.

**Key Points:**

***Question*** *BRAF/MEK and immune checkpoint inhibitors have revolutionized melanoma treatment but are associated with various side effects, yet data regarding the development of mesenteric panniculitis are scarce*.

***Findings*** *BRAF/MEK inhibitor treatment is associated with a significantly higher rate of mesenteric panniculitis compared to immune checkpoint inhibitor treatment in advanced melanoma*.

***Clinical relevance*** *BRAF/MEK inhibitor-treated patients are at risk for development of mesenteric panniculitis. As this benign finding can mimic or conceal malignancy, awareness of it is important especially in these patients*.

**Graphical Abstract:**

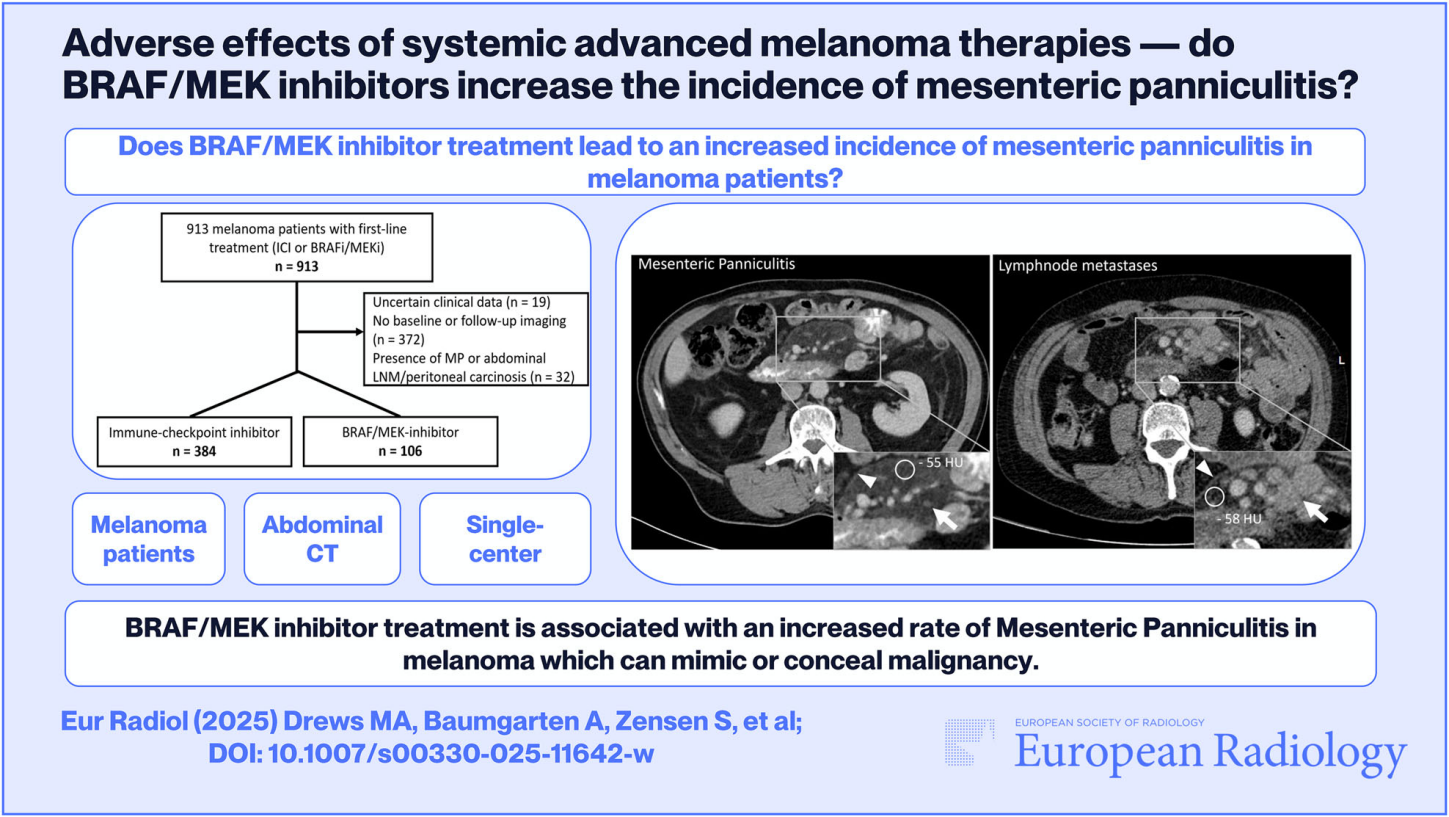

## Introduction

Melanoma is a major health concern in the western world with a rising incidence over the last years [1]. Although accounting for only 1% of all skin malignancies, it is responsible for about 80% of deaths caused by skin cancer [2]. However, a fundamental shift in the therapeutic strategy of advanced-staged melanoma within the last decade has significantly improved patients’ clinical outcomes and survival [3, 4]. Based on a better understanding of cancer genomics and the immune response against cancer, the therapeutic approaches have been changed. Modern therapeutic strategies comprise small-molecule inhibitors (BRAFi/MEKi) on the one hand and antibody-mediated blockade of the cytotoxic T-lymphocyte-associated antigen-4 (CTLA-4) or programmed cell-death protein 1 (PD-1) using immune checkpoint inhibitors (ICI) on the other hand [5, 6]. Approved regimes in BRAF/MEK inhibition include combinations of dabrafenib/trametinib, vemurafenib/cobimetinib and encorafenib/binimetinib, while therapeutic concepts targeting CTLA-4 and PD-1 inhibition comprise nivolumab, pembrolizumab, nivolumab/ipilimumab and relatlimab/nivolumab [6, 7].

Despite major gains in overall survival, these new therapies are associated with a variety of adverse effects that relevantly differ from classical chemotherapy-related toxicities. Both (ICI and BRAFi/MEKi) show a wide spectrum of systemic and organ-related toxicities—most commonly dermatological and gastrointestinal disorders—but prevalence as well as clinical relevance of the individual effects vary between these groups because of different underlying mechanisms [8–10]. An increased rate of panniculitis of the skin following BRAFi/MEKi treatment due to T- and B-cell activation has been reported as a rare side-effect in recent publications [11–14]. Additionally, selected studies also point to an increased incidence of mesenteric panniculitis (MP), but analyses of larger cohorts are scarce [15]. With a prevalence between 0.16 and 3.4% in the general population, MP is a rare but benign disorder characterized by chronic inflammation and fibrosis of mesenteric adipose tissue [16, 17]. Although mostly an asymptomatic incidental finding in CT imaging, MP may lead to clinical symptoms including abdominal pain, bloating/distension, and diarrhea or constipation [17, 18]. Moreover, MP can mimic or even disguise an underlying malignancy [19, 20]. To prevent misinterpretations of these imaging features and to prevent the underdiagnosis of mesenteric malignancies, the aim of this study was to investigate the development of MP following BRAFi/MEKi and ICI treatment in melanoma patients.

## Methods

### Patients

**Fig. 1:**
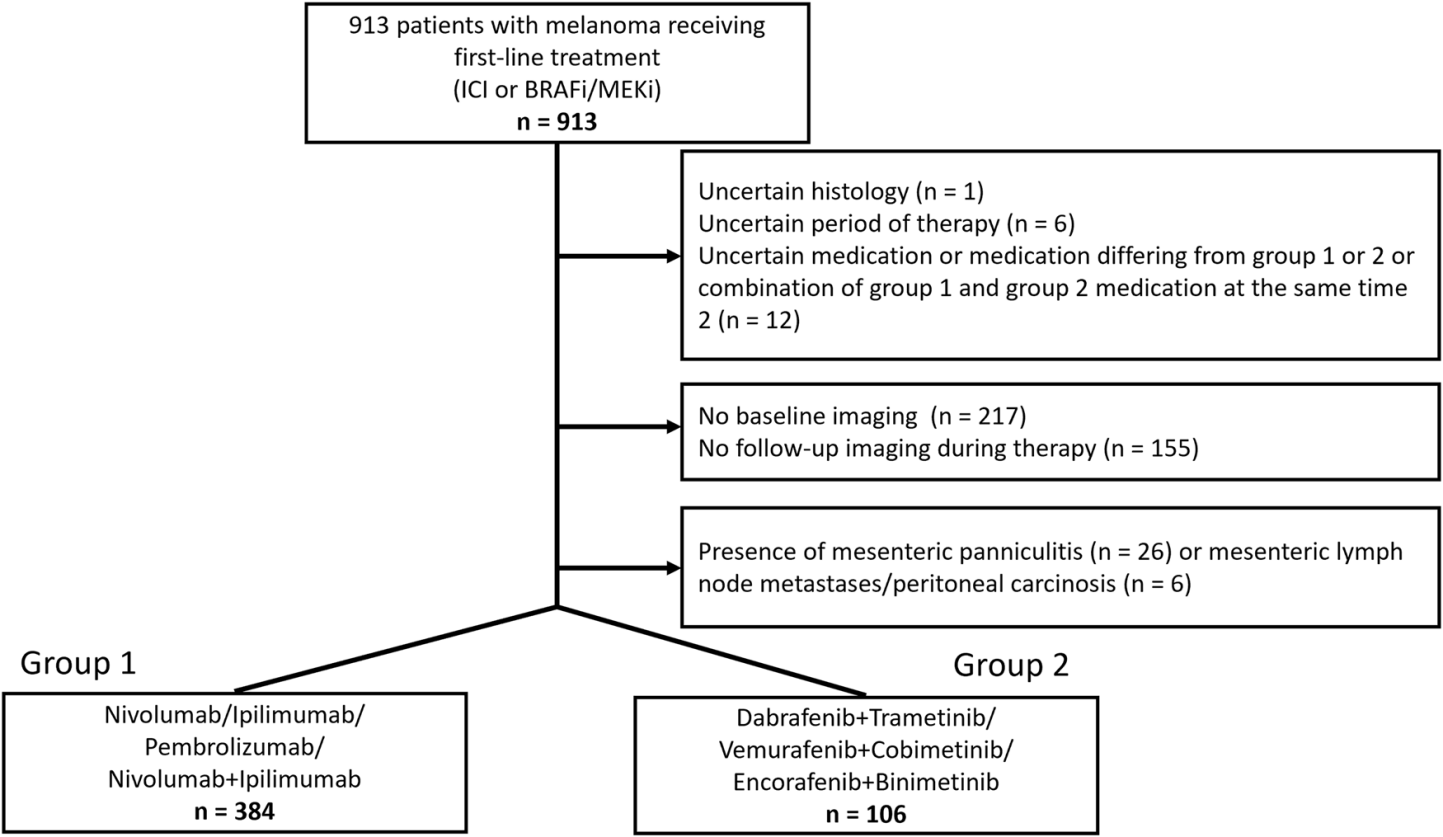
Flow diagram of included patients

For this retrospective study, ethical approval was granted by the Ethics Committee of the Medical Faculty of the University of Duisburg-Essen on 25 July 2024 (20-9190-BO), and the requirement to obtain informed consent was waived. The institutional databases of the Department of Dermatology at the University Hospital Essen were reviewed to identify melanoma patients who were treated with ICI (nivolumab, pembrolizumab, ipilimumab or ipilimumab/nivolumab) or BRAFi/MEKi (dabrafenib/trametinib, vemurafenib/cobimetinib or encorafenib/binimetinib) between 01/01/2010 and 31/12/2023 and received CT imaging before and during therapy. To avoid influence by other drug-based cancer therapies, we included first-line regimes (adjuvant and non-adjuvant situation) only. A total of 913 patients were identified. Among these, 19 patients were excluded because of uncertain histopathological data (*n* = 1), unknown duration of therapy (*n* = 6) or unclear medication (*n* = 12). Additionally, 217 datasets were excluded due to the lack of baseline imaging (full-dose CT < 3 months prior therapy start) and 155 because of missing/unavailable follow-up imaging under therapy (at least 1 full-dose CT > 30 days after therapy start). The presence of MP before therapy started (*n* = 26), as well as abdominal lymph node metastases or peritoneal carcinomatosis (*n* = 6), which could mimic MP, led to the exclusion of 32 further patients. This resulted in a final study cohort of 490 patients (divided into two groups, ICI: 384 patients vs. BRAFi/MEK: 106 patients) (Fig. 1).

### Imaging analyses

CT imaging data (portal venous phase of the whole abdomen) were evaluated for the presence of MP which was defined as an unilocular mesenteric mass characterized by small tissue nodules with inhomogeneous increased density of the adjacent fat (−60 to −40 HU) and surrounding by a pseudo-capsule (< 3 mm, fibrotic rim around the mass) [16, 21]. Additional criteria were a positive fat halo sign—defined as a rim of fat isodense tissue surrounding mesenteric nodules and vessels—and displacement of abdominal structures/organs [16, 21]. In contrast to that, mesenteric lymph node metastasis respectively peritoneal carcinomatosis (PC) was defined as multilocular appearance of mesenteric/peritoneal nodules (> 10 mm), including omental caking, potentially showing stranding of the adjacent fat, displacement of abdominal structures or appearance of ascites [20, 22, 23]. Clinical data, follow-up imaging evaluation of the mesenteric lesions, as well as histopathological data gained from mesenteric biopsy (if available), were used as a composite reference standard for verification of malignancy. Imaging analysis was performed using Centricity Universal Viewer 6.0 (GE HealthCare). The imaging analysis was performed in consensus by two radiologists (M.D. and B.S.) with 3 and 12 years of experience in oncological imaging. The readers were blinded to the applied treatment.

### Statistical analysis

Descriptive statistics were calculated using SPSS 29 (IBM Inc.) and Excel 365 (Microsoft Corp.). Imaging follow-up time was defined as the time between the baseline CT and the last available follow-up CT. Median ± interquartile range (IQR) is presented for non-normally distributed data. Significance was calculated using Fisher’s exact test (two-sided) and Mann–Whitney U test if no normal distribution was observed. Normal distribution was tested using Kolgomorov–Smirnov Test. *p* < 0.05 indicated statistical significance.

## Results

### Patient cohort

**Fig. 2:**
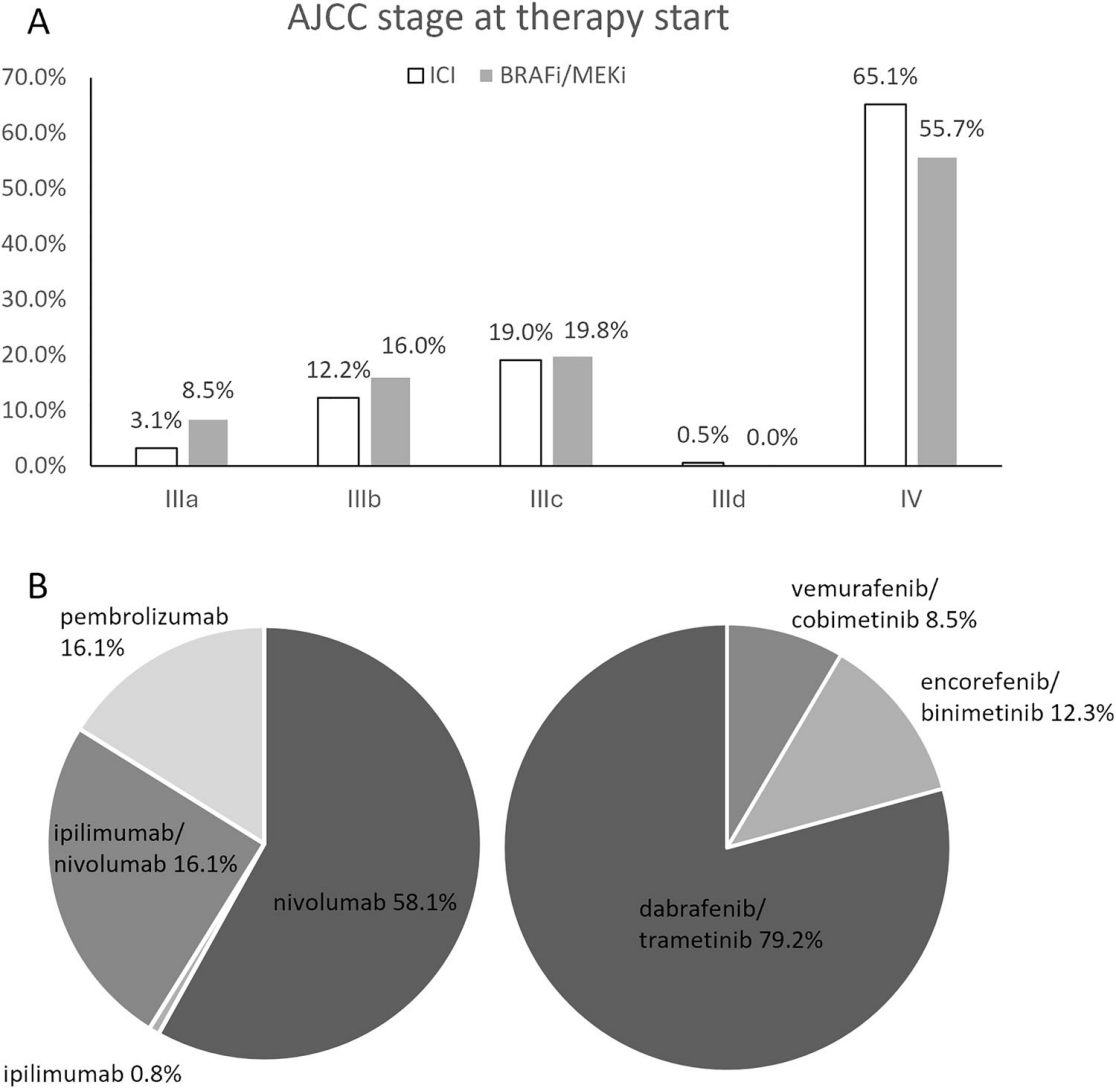
AJCC stage at therapy start with ICI (*n* = 384) or BRAFi/MEKi (*n* = 106) (**A**) and applied therapy regimes (**B**), ICI (left) vs. BRAFi/MEKi (right)

The final study cohort comprised 490 melanoma patients who either received ICI (Group 1, 384 patients) or BRAF/MEKi (Group 2, 106 patients) as first-line treatment (Table 1). Group 1 consisted of 161 female (41.9%) and 223 male patients (58.1%) with an overall median age at therapy start of 62 years (IQR: 51–72 years) compared to 46 female (43.4%) and 60 male (56.6%) median aged 58 years (IQR: 50–68 years) in group 2. Most common tumor stage at therapy start according to AJCC 8th edition was stage IV in group 1 with 65.1% (250/384) as well as in group 2 with 55.7% (59/106) (Fig. 2A). The majority of patients underwent therapy in a non-adjuvant situation in both groups (Group 1: 68.0% [261/384] vs. Group 2: 64.2% [68/106]) compared to the adjuvant situation (Group 1: 31.8% [123/384] vs. Group 2: 35.8% [38/106]). With 58.1% (223/384), nivolumab monotherapy was the predominant therapeutic regime in group 1, followed by ipilimumab/nivolumab combination (25.0%, 96/384), pembrolizumab (16.1%, 62/384) and ipilimumab (0.8%, 3/384) (Fig. 2B). In group 2, 79.2% (84/106) patients were treated with dabrafenib/trametinib, 12.3% (13/106) received encorafenib/binimetinib and 8.5% (9/106) vemurafenib/cobimetinib. Median imaging follow-up from baseline imaging to last available CT was 225 days (IQR: 116–410.25 days) for ICI and 334.5 days (IQR: 173.75–565 days) for BRAF/MEKi with a median number of evaluated CTs of 2 (IQR: 1–3) for ICI and 2 (IQR: 1–5) for BRAFi/MEKi.

**Table 1:** Patient characteristics

	Group 1 (ICI)	Group 2 (BRAFi/MEKi)
Patients	384	106
Sex	161 female (41.9%) 223 male (58.1%)	46 female (43.4%) 60 male (56.6%)
Median age at therapy start	62 years [IQR: 51–72]	58 years [IQR: 50–68]
Therapy situation	non-adjuvant: 261 (68.0%) adjuvant: 123 (32.0%)	non-adjuvant: 68 (64.2%) adjuvant: 38 (35.8%)
Median follow-up (days)	225 [IQR: 116–410.25]	334.5 [IQR: 173.75–565]

### Evaluation of mesenteric panniculitis following treatment

**Fig. 3:**
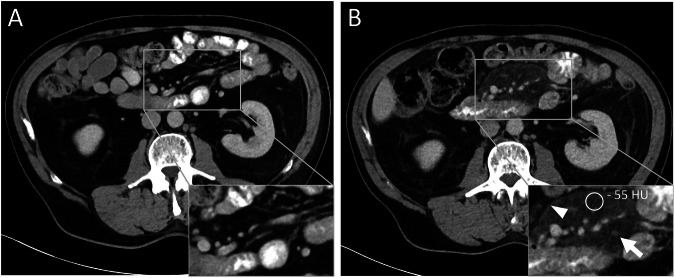
Abdominal CT images of a 72-year-old patient before (**A**) and 234 days after therapy start with dabrafenib/trametinib, showing newly developed mesenteric panniculitis (**B**). Arrowhead indicates pseudo-capsule, arrow points at mesenteric nodules and circular ROI shows increased density of the mesenteric fat. HU, Hounsfield units

**Fig. 4:**
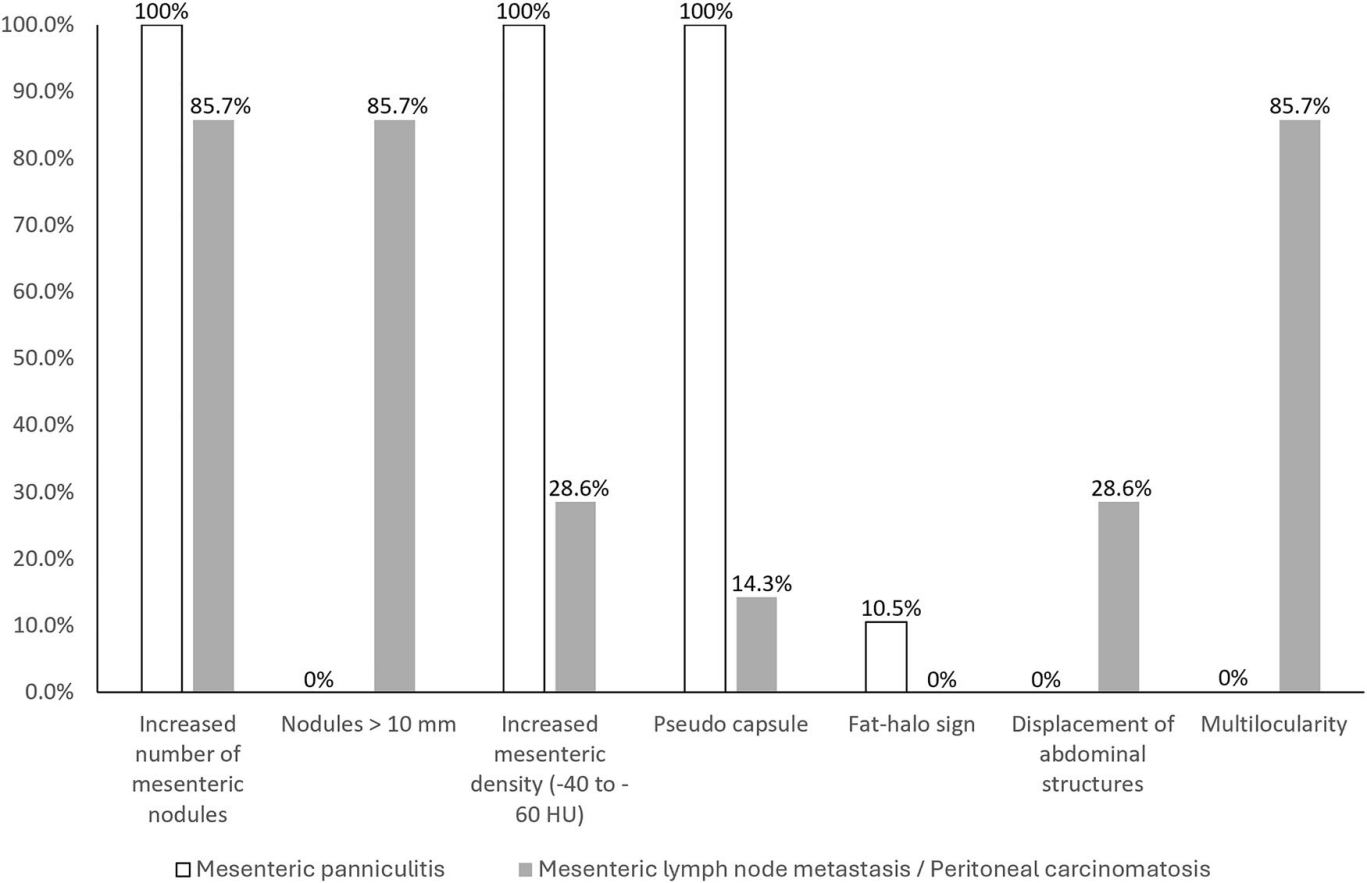
Morphological characteristics of mesenteric panniculitis (*n* = 19) compared to malignancy (*n* = 7)

Incidence of MP was evaluated in CT follow-up examinations compared to the baseline staging before therapy start. A total of 19 patients showed the typical imaging criteria for MP, including the appearance of a mesenteric mass consisting of small tissue nodules with inhomogeneously increased density of the adjacent fat and a surrounding pseudo-capsule (Figs. 3 and 4). A positive “fat halo sign” as an additional MP criterion was only seen in 10.5% (2/19 cases). Displacement of other abdominal structures or organs as a feature of severe MP was not observed.

**Fig. 5:**
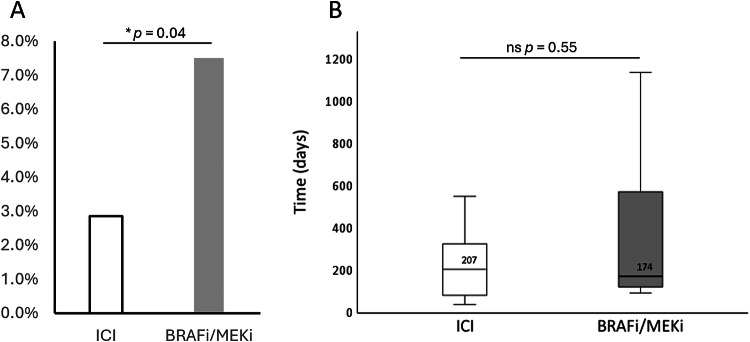
Incidence of mesenteric panniculitis in melanoma patients following first-line treatment with ICI or BRAFi/MEKi (**A**). Time between therapy start and mesenteric panniculitis development in melanoma patients following first-line treatment with ICI or BRAFi/MEKi (**B**). Asterisk indicates significant difference (*p* < 0.05)

Comparing the incidence between both study groups, we observed a significantly higher rate of MP following BRAFi/MEKi compared to ICI treatment: The ICI group showed an MP incidence of 2.9% (11/384) compared to 7.5% (8/106) in BRAFi/MEKi (*p* = 0.04) (Fig. 5A). No relevant differences between both groups were observed comparing the morphology of the MP lesions. Due to the small number of MP cases, no subgroup analysis for the individual substances was performed.

Additionally, the time between therapy start and the first detection of MP in available CT imaging was evaluated. The median time until MP development following ICI was 207 days [IQR: 77–375] and 174 days [IQR: 123–642] in BRAFi/MEKi. This difference was not considered significant (*p* = 0.55) (Fig. 5B).

### Comparison of MP and PC

**Fig. 6:**
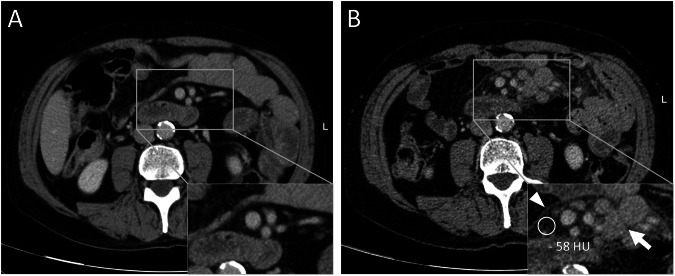
Abdominal CT images of a 71-year-old patient before (**A**) and 340 days after therapy start with Ipilimumab/Nivolumab showing newly developed mesenteric lymph node metastases (**B**). Arrowhead indicates pseudo-capsule, arrow points at mesenteric nodules and circular ROI shows increased density of the mesenteric fat. HU, Hounsfield units

Finally, we evaluated the appearance of mesenteric lymph node metastasis (LNM) respectively PC in our cohort and examined morphological differences compared to MP (Fig. 4). Mesenteric LNM or PC was already present in the baseline staging of six patients who were therefore initially excluded from the evaluation of MP incidence. These patients showed multilocular mesenteric lymph nodes or soft-tissue masses (> 10 mm) that were rarely accompanied by increased mesenteric density (1/6). One patient of the study group (0.2%, 1/490) developed mesenteric LNM during follow-up. In this single case, follow-up CT 695 days after ipilimumab/nivolumab start revealed numerous nodular lesions > 10 mm at the mesenteric root showing increased density of the surrounding fat and the presence of a pseudo-capsule as it was seen in MP (Fig. 6). Histopathological analysis confirmed the diagnosis of melanoma LNM in this case.

In comparison to that, MP in our study was always unilocular with nodules that commonly neither exceeded 10 mm in diameter nor increased significantly in size over time (Fig. 4).

## Discussion

Modern drug-based cancer therapies improved outcome of advanced-staged melanoma patients significantly but are associated with various side effects [3, 8, 9]. MP can mimic or even disguise an underlying malignancy. However, the origin of this commonly encountered benign disease remains unclear. In this retrospective study, we identified a higher rate of MP following BRAFi/MEKi treatment than ICI treatment.

MP is a rare, non-neoplastic disorder with a prevalence between 0.16 and 3.4% in the general population [17]. However, different theories suggest an association with infection, trauma, malignancy, or immunomodulation [17, 24, 25]. Here, a possible link between MP and BRAFi/MEKi treatment has been hinted at by a recent case series [15]. With an MP incidence of 7.5% following BRAFi/MEKi treatment compared to 2.9% in ICI-treated patients, we could verify this observation with our study in a large cohort for the first time and identified MP as a mid-term effect with a median latency to development of more than 6 months after therapy start. The increased MP rate underlines the potential causality between MP and manipulation of mitogen-activated protein kinases (MAPK)-signaling using BRAFi/MEKi. In this context, different hypotheses for MP development are discussed, including MAPK-based pro-inflammatory mechanisms [15, 25–27]. On the other hand, there are also selected case reports describing MP following ICI treatment in different entities [28–30]. However, as the detailed molecular mechanisms of MP development are complex, further preclinical and clinical research might be reasonable to elucidate these.

MP is considered a benign, often asymptomatic condition, but may cause different abdominal symptoms, including abdominal pain, bloating/distension, diarrhea or constipation [18, 31]. However, this finding can indicate an underlying malignancy in 1–4.6% [19, 20, 32–35]. In accordance with the literature, only one patient with LNM was detected in our cohort. In this case, we identified a partial morphological overlap between MP and LNM. Common MP features, including nodular lesions, increased density of mesenteric fat and a pseudo-capsule, can also be found in malignancy [19, 20, 36]. In contrast to MP, LNM as well as PC are commonly multilocular and show progressive lesions > 10 mm that may obstruct other abdominal structures, which are considered worrisome features [20, 22, 36]. But due to the similarities between MP and malignancy, it is crucial in patients at risk for MP development—including BRAF/MEKi treated melanoma patients—to be aware of this condition. In the context of patient management, the MP lesions should be carefully re-evaluated during regular imaging follow-up, not to underestimate or miss malignancy. In case of worrisome features or uncertain cases, biopsy is recommended for differentiating between MP and metastasis. Additional studies also propose F-18-FDG-PET/CT as a helpful tool for differentiating between MP and malignancy by using a standardized uptake value cut-off < 3 for benign mesenteric lesions [19, 22].

### Strengths and limitations

The present study demonstrated a correlation between BRAFi/MEKi treatment and MP development in a large cohort of melanoma patients for the first time. However, a casual relation between these two factors was not investigated in the present study, and further research is necessary. Furthermore, the low total number of MP and the small size of selected subgroups did not allow a sufficient, in-depth subgroup analysis. This is especially important regarding the various BRAFi/MEKi treatment regimens, which are used less frequently in first-line melanoma treatment compared to ICI. Therefore, additional studies with more MP cases are necessary to exclude substance-specific effects. Moreover, the median number of follow-up CTs was low, and the follow-up time showed a large statistical variation, which may have impaired the detection of more panniculitis cases. Although guidelines determine standardized follow-up intervals, the variation in our study could be explained by unintentional extensions of staging intervals during the COVID-19 pandemic and external staging examinations that were unavailable for our analysis. As MP is a chronic condition, differences in follow-up intervals mainly affect the time point of initial MP diagnosis. Given the long overall follow-up timespan of all included patients and the presence of these variations in both groups, its impact on the diagnosis of MP during follow-up imaging seems negligible.

## Conclusion

In conclusion, we detected an increase in MP incidence following BRAFi/MEKi treatment compared to ICI. As MP can mimic or conceal malignancy, MP is an important observation in melanoma patients undergoing repeated staging examinations. In uncertain cases, biopsy is recommended to exclude malignancy.

## Abbreviations

BRAFi/MEKi: “B-rapidly accelerated fibrosarcoma” and “mitogen-activated extracellular signal-regulated kinase” inhibitor
CTLA-4: Cytotoxic T-lymphocyte-associated protein 4
HU: Hounsfield units
ICI: Immune checkpoint inhibitors
LNM: Lymph node metastases
MAPK: Mitogen-activated protein kinases
MP: Mesenteric panniculitis
PC: Peritoneal carcinosis
PD-1: Programmed Cell Death Protein 1
